# Intramyocardial Bridge in Sports Medicine: Proposal of a Possible Follow-Up Strategy in Asymptomatic Athletes

**DOI:** 10.3390/jfmk11020134

**Published:** 2026-03-24

**Authors:** Roberto Palazzo, Melissa Orlandi, Federico Fu, Vittorio Bini, Laura Stefani

**Affiliations:** 1 Sports Medicine Centre, Clinical and Experimental Medicine, University of Florence, 50134 Florence, Italy; roberto.palazzo@unifi.it (R.P.); dott.melissaorlandi@gmail.com (M.O.); federicfu@gmail.com (F.F.); 2 Department of Medicine, Section of Internal Medicine, Endocrinology and Metabolism, University of Perugia, 06126 Perugia, Italy; binivittorio@gmail.com

**Keywords:** intramyocardial bridge, CPET, Echocardiography, SPECT, sports medicine, twist, strain, GLS, COCIS

## Abstract

**Background:** Intramyocardial bridge (MB) is a coronary anomaly characterized by a segment of the artery tunneling within the myocardium. While often asymptomatic, it may lead to ischemic events. Despite traditional disqualification from competitive sports, 2023 guidelines now permit participation for athletes with MBs that do not meet specific high-risk morphological criteria. This study aims to evaluate a novel combined provocative test, integrating Cardiopulmonary Exercise Testing (CPET) and stress echocardiography for the assessment of myocardial deformation (twist), to assess the functional impact of MB in asymptomatic athletes. **Methods:** This cross-sectional case–control study included 18 participants (nine cases with “significant” MB diagnosed via Computed Tomography (CT) coronary angiography and nine healthy, trained controls), aged 18–78 years. All subjects underwent evaluation at our facility for competitive certification. Assessment protocols included resting echocardiography, Global Longitudinal Strain (GLS), and Cardiopulmonary Exercise Testing (CPET) to quantify exercise capacity and dynamic myocardial function. **Results:** No significant differences in echocardiographic parameters were observed between groups at rest. However, during exercise, athletes with MB demonstrated a significant reduction in GLS and ventricular twist compared to the control group. These findings indicate a notable loss of apical reserve in the MB cohort during physical stress. **Conclusions:** The integration of CPET and myocardial deformation analysis provides an effective diagnostic tool for identifying functional impairment in asymptomatic athletes with MB. This combined approach offers a superior follow-up strategy for managing athletes who may be at risk for ischemic events despite lack of clinical symptoms.

## 1. Introduction

Intramyocardial bridge (MB) is a specific coronary anomaly, often asymptomatic, but potentially associated with inducible ischemic events [1]. It is largely considered a benign variant; however, in sports medicine, and particularly in the context of Italian sports eligibility, it has been a cause for disqualification for many years, as suggested by the COCIS committee established to define Italian guidelines and the Cardiological Protocols for sports eligibility in 2017 [2]. The relevance of and attention to this topic are due to the demonstration of a potential link with acute events, as in the case of MINOCA (Myocardial Infarction with Non-Obstructive Coronary Arteries) [3]. With the revision of COCIS in 2023, the approach has changed. MB forms that are not considered significant in terms of morphology (depth and extension) may be compatible with eligibility. However, significant forms (depth > 3 mm and extension > 10 mm) must undergo specific testing to demonstrate the absence of reduced perfusion and any potential impact on the hemodynamic component.

The most commonly used tests to identify inducible ischemia are CPET, physical stress echo, myocardial scintigraphy with exercise (SPECT), and coronary angiography. All these are included in the sports medicine protocol suggestions; however, they are not proposed in a sequential manner, as they are considered equally important, without any priority, and therefore without any substantial difference in their specific contribution to prognostic value.

Previous studies have reported the role of nuclear scintigraphy stress testing as consistent and indicative of the presence of an MB in the left anterior descending (LAD) coronary artery [4,5], although with low specificity in identifying a simple perfusion deficit [6,7] due to the “Venturi mechanism” in the first phase of diastole. On the contrary, the cardiopulmonary test is often used in cases of suspected reduced myocardial capacity [8], despite the test itself not offering a specific evaluation of segmental myocardial behavior. In this context, echocardiographic deformation parameters, like myocardial strain analysis and the study of torsion, could be helpful in highlighting potential myocardial dysfunction.

The aim of this study was to evaluate the functional and clinical impact of integrating Cardiopulmonary Exercise Testing with stress echocardiographic myocardial deformation analysis in asymptomatic athletes with MB to highlight different degrees or a major grade of severity in terms of cardiovascular risk linked to the sports activity. 

## 2. Materials and Methods

### 2.1. Study Population and Design

This is a cross-sectional case–control study that included a sample of 18 participants (9 cases and 9 controls), aged 18 to 78 years, who underwent the competitive certification process at our center between 2022 and 2024. This study was conducted following the Declaration of Helsinki and in accordance with privacy protection regulations. All subjects gave formal consent to participate in the investigation. The data were retrieved from the database of the Sports Medicine Center and were acquired during routine sports eligibility assessments.

This study is part of a larger protocol approved by the Ethics Committee for Clinical Trials in Padova (protocol code 5254/AO/21). The involvement of the University of Florence began in 2022.

The case group, consisting of 9 athletes (mean age 41.6 years), had a confirmed diagnosis of a “significant” MB based on at least one morphological criterion (extension or depth). The control group consisted of 9 healthy, trained subjects with a mean age of 27.7 years. The case group had previously undergone CT coronary angiography due to ST-T alterations suggestive of ischemia during exercise testing in prior assessments. The mean age at diagnosis was 39.67 years. All subjects were male, Caucasian, and asymptomatic for chest pain, dyspnea, and syncope. Seven out of nine athletes had a bridge located on the left anterior descending artery (LAD, segments II–III), while two had a bridge on the right coronary artery (RCA).

Inclusion criteria

- A confirmed coronary anomaly diagnosis (MB).
- A uniform level of training and/or physical activity between groups, estimated using the IPAQ (with IPAQ > 2500 METS/week).
- Willingness to participate in the required diagnostic and physical tests.

Exclusion criteria

- Severe cardiac diseases (e.g., advanced heart failure), cardiological symptoms, or conditions that would preclude eligibility.
- Severe chronic renal insufficiency.
- Use of medications that affect cardiovascular parameters (e.g., beta-blockers).
- Inability to perform the Cardiopulmonary Exercise Test (CPET) due to physical or neurological limitations.

According to the study protocol, and prior to conducting the CPET, all subjects were screened for demographic characteristics and cardiovascular risk factors. The case group had also previously undergone myocardial scintigraphy to either demonstrate or exclude coronary flow perfusion deficits induced by physical exercise. A specific 24-h Holter ECG (3 leads) was only performed for the case group (MB).

The initial investigation showed that the case population had a higher cardiovascular risk profile (Table 1), particularly regarding lifestyle factors, compared to the controls.

### 2.2. Methods

#### 2.2.1. Standard Echocardiography

Following the study protocol, all subjects underwent 2D transthoracic echocardiography (TTE). The main systolic and diastolic parameters were measured in accordance with the ASE guidelines [9]. For the standard parameters, the following measurements were considered: left ventricle end-diastolic diameter (LVEDD), the interventricular septum (IVS) and posterior wall (PW) of the left ventricle, left ventricle (LV) cardiac mass, and indexed left atrial volume (LAVI). Regarding diastolic function, the mitral inflow pattern (E/A ratio, deceleration time) and pulsed-wave Tissue Doppler Imaging (TDI) of the septal and lateral mitral annulus with the E/e′ ratio were considered. The average mitral E/e′ ratio was calculated and regarded as a reliable index of left atrium (LA) filling pressures. Diastolic function was classified as normal or abnormal: impaired relaxation (grade 1), pseudo-normal (grade 2), or restrictive (grade 3).

#### 2.2.2. Strain Analysis

2D echocardiography included the specific images for post-processing evaluation of myocardial deformation parameters such as Global Longitudinal Strain (GLS) and ventricular twist. These two specific parameters were considered the main expressions of the longitudinal and circumferential contribution of the myocardial fibers during the contraction. The acquisition and analysis was obtained using the speckle tracking method. For the correct acquisition of cycles during CPET, the heart rate range for capturing images was established between the 1st and 2nd thresholds, as measured by CPET. This choice was made to reduce potential errors caused by artifacts due to elevated heart rates. Dedicated software included in the MyLab Echo system (XStrain™, ESAOTE, Genoa, Italy) was used for specific post-processing analysis. According to the criteria established in the EACVI/ASE consensus document [10], it was possible to obtain the average myocardial deformation by acquiring images over a full cardiac cycle at a high frame rate. Based on the EACVI/ASE guidelines and estimated data reproducibility [11], normal GLS values were considered to range from −24% to −16% and normal left ventricular twist values ranged from 10° to 20°.

#### 2.2.3. Cardiopulmonary Exercise Test (CPET)

The CPET was conducted based on international guidelines [12,13,14] using an electromagnetically braked cycle ergometer (Ergoline) and a specific gas measurement machine (COSMED Quark CPET, Albano Laziale, Rome, Italy). Measurements of VO2 max, peak oxygen pulse, and the VE/VCO2 slope were recorded. ECG parameters were monitored throughout the test. Each participant was instructed to avoid strenuous physical activity the day before the test and to abstain from consuming solid foods or carbohydrate-rich drinks for three hours prior to the assessment.

The test was performed in the morning under controlled conditions (temperature: 18–24 °C; humidity: 30–60%). The ramp protocol was tailored based on gender and body composition, aiming for volitional exhaustion between 8 and 12 min. An oro-facial mask connected to a gas-measuring device was used. Exhaled CO_2_ and O_2_ consumption were measured breath by breath. A small workload increment (1, 2, or 5 watts) was set for each ramp to achieve a linear increase in load and a more physiological response. After 3 min of warm-up cycling without load at 50 rpm, participants cycled at a cadence between 60 and 80 rpm until exhaustion.

The test concluded when the participant could no longer maintain the required cadence despite verbal encouragement. The test was considered maximal if at least two of the following criteria were met:

1. Respiratory Exchange Ratio (RER) > 1.10.
2. Maximum heart rate > 85% of the age-predicted maximum.
3. A plateau in oxygen consumption (increase < 150 mL/min) in the last 30 s.

The test was stopped early in the presence of cardiovascular signs or symptoms (complex ventricular arrhythmias, drops in systolic blood pressure, dizziness, etc.). Continuous monitoring included a 12-lead ECG and oxygen saturation. Parameters measured included VO2, VCO2, tidal volume (VT), respiratory frequency (RF), minute ventilation (VE), heart rate (HR), and workload (WR). The lactate threshold was determined using the V-slope and ventilatory equivalent approach. Other variables analyzed included the VO2/HR relationship (an indirect measure of stroke volume), the VO2/W slope (circulatory efficiency), and the product of peak VO2 and systolic blood pressure (circulatory power).

#### 2.2.4. Specific Exams for the Case Group

- The 24-h Holter ECG: To analyze the presence of arrhythmias and repolarization abnormalities.
- Myocardial Scintigraphy (Rest and Stress): To assess myocardial perfusion.
- Coronary CT (CCTA): For the detailed analysis of coronary anomalies.

During the CPET, between the first and second thresholds (identified by the VO2 max graph and/or ventilatory threshold), cineloop images were acquired for subsequent analysis of deformation parameters, specifically GLS and twist.

### 2.3. Study Design

This was a cross-sectional case–control study aimed at evaluating both athletes with an MB and controls. The study assessed their training levels along with regional and global cardiovascular function using the diagnostic tools mentioned above.

### 2.4. Study Phases

**Figure 1:**
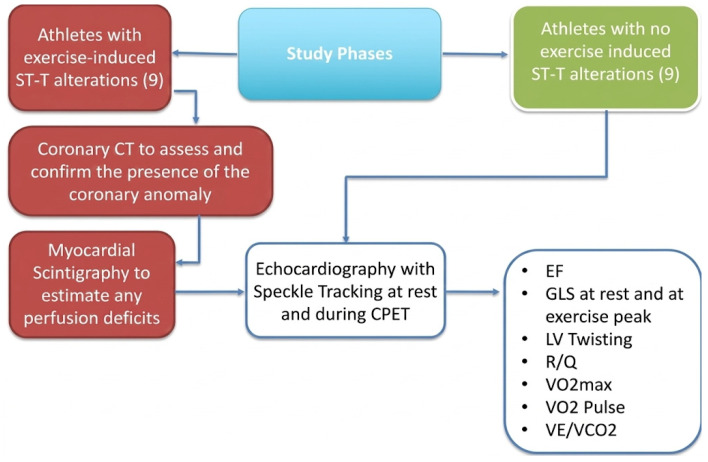
Study phases.

Participants were selected from the sports medicine center based on the inclusion and exclusion criteria and underwent evaluations as shown in Figure 1. Clinical, anthropometric, and diagnostic parameters were recorded. Each participant completed the following:

- Coronary CT (CCTA) for cases to assess and confirm the presence of the coronary anomaly in the presence of ST-T alterations suggestive of exercise-induced ischemia.
- The 24-h Holter ECG to assess the progression of ST-segment changes and any associated asymptomatic arrhythmias.
- Myocardial scintigraphy to estimate any perfusion deficits.
- Echocardiography with Speckle Tracking at rest and during CPET.
- Cardiopulmonary Exercise Test (CPET) to evaluate exercise capacity and respiratory function.

The data were analyzed in accordance with the international recommendations of the ESC (European Society of Cardiology) and, specifically, the Italian national guidelines for competitive sports eligibility, COCIS in 2023. This dual approach ensured compliance with both international clinical standards and specific national regulatory requirements for sports certification.

### 2.5. Statistical Analysis

Descriptive statistics were used to summarize clinical and demographic characteristics. To evaluate the association between myocardial deformation parameters and the presence of MB and to identify potential functional indicators, inferential statistical analyses were performed. The Shapiro–Wilk test was used to assess the normal distribution of the variables. Due to their skewness, the Mann–Whitney U test was applied to compare non-normally distributed continuous and discrete variables. The Chi-square test with Yates’ continuity correction and Fisher’s exact test was used to compare categorical variables. All statistical analyses were performed using IBM-SPSS^®^ version 26.0 (IBM Corp., Armonk, NY, USA, 2019). In all analyses, a two-tailed *p*-value < 0.05 was considered statistically significant.

## 3. Results

### 3.1. SPECT

Myocardial perfusion tomography (SPECT) was performed exclusively in the case group to assess the presence of myocardial ischemia in subjects diagnosed with a significant MB. The results were analyzed using the standard 17-segment ventricular model, according to the QPS report, to determine the distribution and frequency of exercise-induced ischemia.

1. Mild Ischemia (Value 1): Mild ischemia was detected in the following segments and coronary territories:
  ○ Segments 4 and 10: Exercise-induced ischemia in Patient 3.
  ○ Segment 8: Exercise-induced ischemia in Patient 7.
  ○ Segments 11 and 12: Exercise-induced ischemia in Patient 9.
2. Moderate Ischemia (Value 2): Moderate ischemia was observed exclusively under stress in Segment 16 in Patient 9.
3. Absence of Ischemia (Value 0): The majority of the analyzed segments (148 out of 153, or 96.73%) showed no evidence of ischemia, either at rest or under stress.
  Distribution of ischemic segments by patient:
  - Patient 1: No ischemia detected.
  - Patient 2: No ischemia detected.
  - Patient 3: Two ischemic segments (RCA territory, mild under stress).
  - Patient 4: No ischemia detected.
  - Patient 5: No ischemia detected.
  - Patient 6: No ischemia detected.
  - Patient 7: One ischemic segment (LAD territory, mild under stress).
  - Patient 8: No ischemia detected.
  - Patient 9: Three ischemic segments (two LCx: one mild and one moderate under stress; one RCA: mild under stress).

These results reflect the pathophysiology of the MB, which typically limits coronary blood flow under conditions of increased hemodynamic demand. The presence of moderate ischemia, observed in only one patient (Patient 9), highlights the variability in the hemodynamic impact among the subjects analyzed.

### 3.2. Standard Echocardiography

All data are expressed as mean ± SD. The standard resting echocardiographic analysis showed no significant differences between the case and control groups for most parameters. The ejection fraction (EF) was slightly lower in the cases (65.1% ± 4%) compared to the controls (67.8% ± 6%), with a difference of −2.7% (*p* = 0.307). Regarding the thickness of the interventricular septum (IVS) and the posterior wall (PW), the average values in the cases were 9.4 ± 1 mm and 9.2 ± 1.1 mm, respectively—slightly higher than in the controls (9.20 ± 0.5 mm and 9.1 ± 0.6 mm), with *p* = 0.564 for both comparisons. The left atrial (LA) diameter was smaller in the cases (32.1 ± 2.8 mm) compared to the controls (34.1 ± 2.4 mm), with a difference of −2.0 mm (*p* = 0.145). Finally, left ventricular end-diastolic volumes (LVEDVs) showed no significant difference, with slightly lower average values in the cases (49.1 ± 3.9 mL) compared to the controls (50.2 ± 1.6 mL), *p* = 0.690. These data indicate that the two study populations are homogeneous regarding standard echocardiographic parameters, including Left Ventricular Ejection Fraction (LVEF), which remained within normal limits for both groups.

### 3.3. Analysis of Strain and Twist Parameters

Data related to strain and twist parameters, both at rest and during stress, were analyzed in both groups.

- GLS (Global Longitudinal Strain): At rest, cases showed a mean value of −18.1% ± 3.2% vs. controls (−18.8% ± 1.4%) with no significant difference, *p* = 0.756. However, during stress, the difference became significant, with mean values of −15.4% ± 4.3% in cases and −21.2% ± 2% in controls (*p* = 0.003). This suggests a reduction in longitudinal contractile capacity (reduced longitudinal contribution) in cases under physical stress.
- Circumferential Strain: The apical reserve contribution, estimated via global circumferential strain (GCS) at the apex, was similar between groups at rest (cases: −6.28% ± 3% controls: −6.3% ± 2.1%), *p* = 0.825. However, mean values for basal circumferential strain in the cases were more negative (−6% ± 2.2%)—and thus more pronounced—than in the controls (−4.4% ± 1.8%), *p* = 0.102. During stress, apical rotation (GCS) significantly increased in the controls but not in the cases. This suggests a loss of the characteristic physiological “athlete-like” behavior in subjects with a myocardial bridge.
- Ventricular Twist: At rest, twist was slightly greater in cases (10.6° ± 3.5°) compared to controls (9.5° ± 2.2°), *p* = 0.724. However, this behavior reversed significantly under stress, with mean values of 8.3° ± 1.9° in the cases and 16.9° ± 3.5° in the controls, *p* < 0.0001. This indicates a failure to mobilize apical reserve in subjects with a myocardial bridge. Upon analyzing myocardial deformation during stress, significant differences were found in the apical contribution: while twist increased in controls, it decreased in the cases. This atypical finding is potentially related to reduced subendocardial perfusion during exercise, leading to a functional deficit in the territory supplied by the bridged artery.

### 3.4. Cardiopulmonary Exercise Testing (CPET)

Data collected during CPET showed significant differences between cases and controls. VO2 max, indicative of cardiorespiratory capacity and exercise performance, was significantly lower in cases (36.1 ± 5 mL/kg/min) compared to controls (53.4 ± 9.4 mL/kg/min), with a difference of −17.3 mL/kg/min, *p* = 0.001. The oxygen pulse, reflecting oxygen transport efficiency and stroke volume during exercise, was also lower in cases (14.5 ± 6.5 mL/beat) vs. controls (20.5 ± 4.2 mL/beat), *p* = 0.038. In contrast, the VE/VCO2 slope, representing ventilatory efficiency, was slightly higher in cases (24.6 ± 2.8) compared to controls (24.3 ± 1.7), *p* = 0.627. These latter data are still under investigation and are currently considered outside the main scope of the present study.

### 3.5. Electrocardiographic (ECG) and Holter Abnormalities

Abnormalities were observed only in the case group, both during maximal exercise testing and Holter monitoring:

- ST-segment depression > 1 mm: Four cases (44.4%).
- ST-segment depression < 1 mm: Four cases (44.4%).
- ST-wave normalization during recovery: Four cases (44.4%).
- Inferolateral alterations: Four cases (44.4%).
- Right Bundle Branch Block (RBBB): Six cases (66.7%).
- Supraventricular ectopy (>500/24 h): Six cases (66.7%).
- Simple ventricular arrhythmias: Six cases (66.7%).
- Complex ventricular arrhythmias: One case (11.1%).

In the control group, no significant abnormalities were observed during the maximal exercise test (ST-segment alterations and arrhythmias were absent). As per the study protocol, Holter monitoring was not performed on the control group.

### 3.6. Correlations Between Myocardial Deformation and CPET in the Global Population and Cases

**Figure 2:**
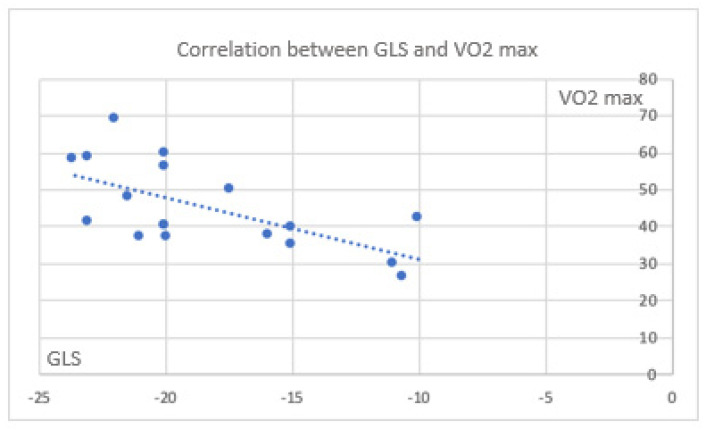
Correlation between GLS and VO_2_ max in the global population (rho = −0.622; *p* = 0.006).

**Figure 3:**
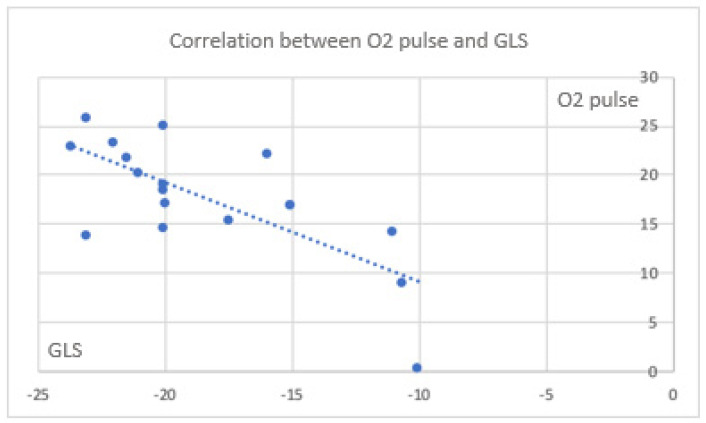
Correlation between O_2_ pulse and GLS in the global population (rho = −0.632; *p* = 0.005).

Color mapping highlights correlations between CPET parameters and those measured during stress echocardiography, with a focus on deformation parameters in the global population investigated. All echocardiographic markers of myocardial deformation correlated significantly with exercise capacity parameters obtained from CPET, confirming the robust impact of the two methods. In the cases, the correlation between CPET data and deformation parameters indicated a trend toward a simultaneous maintenance of exercise capacity and GLS, despite the latter remaining less significant (Figure 2 and Figure 3).

**Figure 4:**
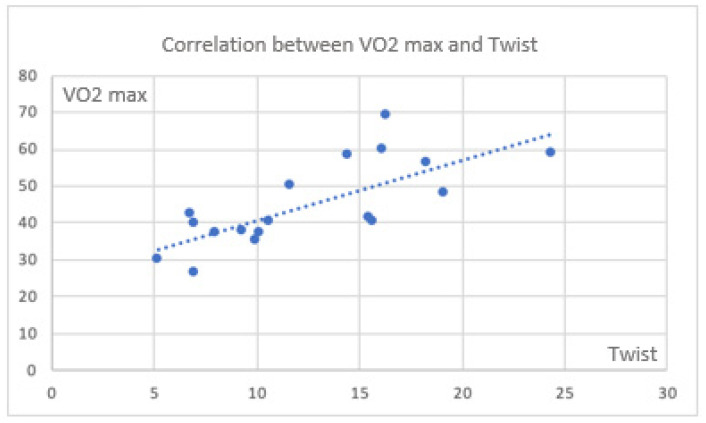
Correlation between VO_2_ max and twist in the global population (rho = 0.769; *p* < 0.0001).

**Figure 5:**
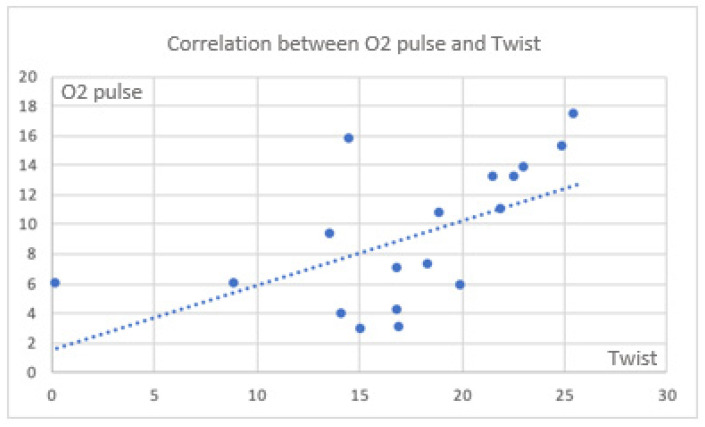
Correlation between O_2_ pulse and twist in the global population (rho = 0.680; *p* = 0.002).

There were also significant correlations between CPET parameters and twist (Figure 4 and Figure 5). 

**Figure 6:**
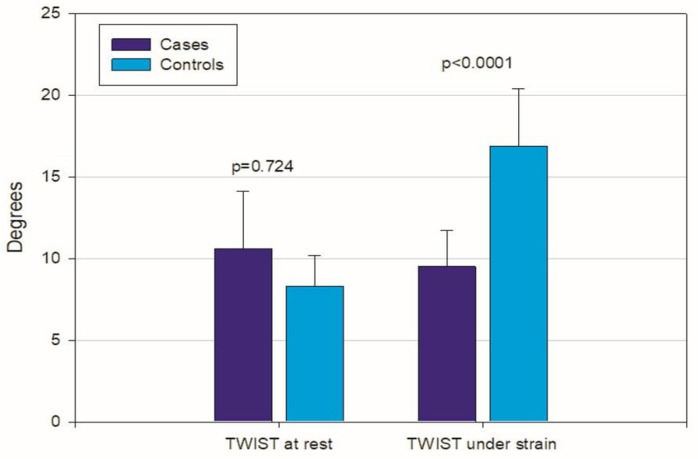
Average values of twist at rest (left) and twist under strain (right), indicating cases have a significant reduction compared to controls, despite being within the normal range.

Considering the torsion parameter as an expression of apical reserve and its potential role in maintaining an increased contribution to the global myocardial contraction, the correlation between VO2 max and twist is lost in some cases. On the contrary, this correlation is clearly evident in the control group (Figure 6). This aspect could be, in the first line, considered as a specific behavior of cases. 

Furthermore, during exercise, a significant positive correlation between circumferential strain at the apex and the oxygen pulse is maintained in the control group, whereas this relationship appears altered in the case group. These findings suggest that the mechanical impairment caused by the myocardial bridge during high-intensity exercise may disrupt the physiological coupling between myocardial deformation and cardiorespiratory efficiency.

In parallel, comparing the deformation parameters in the two groups, the data obtained suggest a specific pattern in subjects with MB, which becomes evident during the exercise test. In particular, the pattern of the twist parameter during exercise is suggestive of an absence of normal recruitment of the apical reserve in cases. There is evidence of a significant reduction in both twist and GLS during exercise as being an expression of potential loss of apical contribution.

In summary the specific correlation of the two parameters derived from different investigations (CPET and echocardiography) highlights the importance of studying these events in the acute phase of exertion to highlight an eventual lack of correlation with VO2 max and oxygen pulse. In contrast, the controls, who maintain their apical reserve, show a significant and positive correlation in both parameters during exercise.

## 4. Discussion

The MB refers to an anomalous course of the coronary arteries, which has been for a long time traditionally considered incompatible with eligibility for elite sports, particularly under Italian regulations, COCIS in 2017. The potential impairment of coronary blood flow, especially during the systolic–diastolic phases of exercise, has made it a key subject of study in recent years [15,16,17]. Despite this, a recent review in sports cardiology, incorporating an anatomical classification of the myocardial bridge in terms of morpho-functional aspects, has led to a broader inclusion of affected individuals. Specifically, those who do not present both of these critical characteristics of the anomaly (depth > 3 mm; length > 10 mm; and other so-called “significant” features [18]) may still be eligible for sports activities. An accurate description by TC angiography of the characteristics of the coronary vessels is therefore determinant in order to exclude the forms absolutely at high risk from a restricted group afferent to a gray zone worthy of further investigation. 

In addition, the appropriate follow-up of athletes—especially those in the “gray zone” of the anatomical classification—and their eventual inclusion in regular training have not yet been thoroughly investigated. This is particularly relevant when no clinical symptoms are present, even during exercise. In such cases where only one of the morphological criteria is met but is accompanied by ECG changes, ST-T patterns suggestive of ischemia may emerge. These factors can lead to interpretative and management uncertainties, especially during the annual follow-up of athletes seeking sports eligibility, particularly for individuals over the age of 35 or those with additional comorbidities.

Therefore, it is crucial in sports medicine to optimize testing methods that, following a definitive morphological diagnosis—especially in borderline cases—can confirm the absence of ischemia in an outpatient setting. In this context, an integrated dynamic test providing additional information becomes indispensable. In line with previous studies [15], the combination of CPET supported by dynamic assessments of deformation parameters at rest and during exercise (e.g., GLS and twist) could offer a valuable approach for this group, including during follow-up.

In particular, dynamic CPET integrated with myocardial deformation data provides enhanced interpretative possibilities. GLS and twist during exercise show a trend of correlation with VO2 max and oxygen pulse, demonstrating that the longitudinal and torsional mechanical functions of the heart are key indicators of a preserved aerobic capacity and cardiac efficiency. Additionally, circumferential strain at the apex and base during exercise shows strong correlations with VE/VCO2 and VO2 max, emphasizing the role of the heart’s circumferential function in ventilation and aerobic capacity.

The absence of significant differences between the two groups at rest further supports the homogeneity and robustness of the data, especially studied in a dynamic condition. The results indicate that, while the cases do not differ from the controls during the resting phase, they display a unique pattern during exercise, marked by a loss of the apical rotation contribution, particularly after the first threshold. It is well known that the circumferential role in the apex zone represents a major involvement in the LV ejection fraction. This finding correlates with a mild reduction in longitudinal strain. In contrast, controls exhibit a significant increase in both parameters. Despite these differences, the cases remain within normal functional limits, suggesting that their ventricular function is preserved and that they may still be eligible for sports.

The current scientific literature is notably scarce regarding the long-term functional follow-up of athletes with MB, especially those who do not meet all high-risk morphological criteria but show subtle ECG alterations. Most existing studies focus on anatomical diagnosis or symptomatic patients, leaving a significant data gap concerning the physiological evolution of myocardial deformation during exercise in asymptomatic individuals. Our study highlights the need for more robust, multicenter data to validate integrated protocols like the one proposed here, which could eventually standardize the management of these athletes in clinical practice. 

## 5. Limitation of the Study

Despite the promising results, this study has some limitations. First, the sample size is relatively small (n = 18), which may limit the generalizability of the findings. However, it should be noted that while MB has a reported prevalence of 19–22% in the general population via CCTA [15], the ‘significant’ forms or those falling into the ‘gray zone’ (requiring deep functional investigation in asymptomatic athletes) represent a much smaller and more specific subgroup. Additionally, the wide age range of our participants (18–78 years) reflects the diverse population of athletes seeking competitive certification but may introduce variability. This heterogeneity is justified by the relatively low clinical expression of this abnormality in the active athletic population, making it challenging to recruit large, age-homogeneous cohorts of asymptomatic athletes with confirmed significant MB.

## 6. Conclusions

The results of this pilot study suggest that the integration of Cardiopulmonary Exercise Testing (CPET) and stress echocardiographic myocardial deformation analysis represents an effective diagnostic approach for the functional evaluation of athletes with intramyocardial bridges (MBs), especially those in a gray zone of coronary morphological anomaly. Our findings highlight that the reduction in ventricular twist and Global Longitudinal Strain (GLS) during exercise can identify early myocardial functional impairment, even in asymptomatic subjects. While these results are based on a limited sample size and require further validation in larger cohorts, this combined protocol offers a valuable tool for sports medicine physicians to improve risk stratification. This approach could be fundamental in clinical decision-making regarding competitive sports eligibility, potentially allowing for safer participation and more tailored follow-up strategies for athletes with MB. The proposed protocol, which combines two non-invasive examinations performed during exercise, represents a valid supportive tool for the long-term follow-up of these athletes during annual sports medicine evaluations.

## Figures and Tables

**Table 1:** Cardiovascular risk profile in case and control groups.

	Age	BMI	Hypertension	Smoking	IPAQ	Total Cholesterol >240 mg/dL	Alcohol Consumption (>2 units/day)
Case group (9)	40 ± 19	22.83 ± 2.74	0 (100%)	3 (33.3%)	>2500 METS/week	2 (22.1%)	2 (22.1%)
Control group (9)	27.7 ± 1.8	23.47 ± 1.81	0 (100%)	0 (100%)	>2500 METS/week	0 (100%)	0 (100%)
*p*-value	0.233	0.402	-	0.206	-	0.453	0.453

## Data Availability

The original contributions presented in this study are included in the article. Further inquiries can be directed to the corresponding author.

## Abbreviations

The following abbreviations are used in this manuscript:

CO_2_	Carbon dioxide
CPET	Cardiopulmonary Exercise Test
CT	Coronary Tomography
EF	Ejection fraction
GLS	Global Longitudinal Strain
HR	Heart rate
IPAQ	International Physical Activity Level
IVS	Interventricular septum
LA	Left atrium
LAD/RAD	Left/right anterior descending coronary
LVEDD	Left ventricle end-diastolic diameter
MB	Intramyocardial bridge
MINOCA	Myocardial Infarction with Non-Obstructive Coronary Arteries
O_2_	Oxygen
PW	Posterior wall
RER	Respiratory Exchange Ratio
RF	Respiratory rate
TDI	Tissue Doppler imaging
VCO2	Carbon dioxide production
VE	Minute ventilation
VO2	Oxygen consumption
VO2 peak	Maximum oxygen consumption
VO2/HR	Oxygen pulse
VO2/W	Relationship between oxygen consumption and workload
VT	Tidal volume
WR	Workload
